# Microchannel-embedded implantable device with fibrosis suppression for prolonged controlled drug delivery

**DOI:** 10.1080/10717544.2022.2032873

**Published:** 2022-02-11

**Authors:** Han Bi Ji, Jae Young Hong, Cho Rim Kim, Chang Hee Min, Jae Hoon Han, Min Ji Kim, Se-Na Kim, Cheol Lee, Young Bin Choy

**Affiliations:** a Interdisciplinary Program in Bioengineering, College of Engineering, Seoul National University, Seoul, Republic of Korea; b Institute of Medical & Biological Engineering, Medical Research Center, Seoul National University, Seoul, Republic of Korea; c Department of Pathology, Seoul National University College of Medicine, Seoul, Republic of Korea; d Department of Biomedical Engineering, Seoul National University College of Medicine, Seoul, Republic of Korea

**Keywords:** Controlled drug delivery, diclofenac, fibrosis, implantable device, microchannel

## Abstract

For the prolonged, controlled delivery of systemic drugs, we propose an implantable drug-delivery chip (DDC) embedded with pairs of a microchannel and drug-reservoir serving as a drug diffusion barrier and depot, respectively. We pursued a DDC for dual drugs: a main-purpose drug, diclofenac (DF), for systemic exposure, and an antifibrotic drug, tranilast (TR), for local delivery. Thus, the problematic fibrotic tissue formation around the implanted device could be diminished, thereby less hindrance in systemic exposure of DF released from the DDC. First, we separately prepared DDCs for DF or TR delivery, and sought to find a proper microchannel length for a rapid onset and sustained pattern of drug release, as well as the required drug dose. Then, two distinct DDCs for DF and TR delivery, respectively, were assembled to produce a Dual_DDC for the concurrent delivery of DF and TR. When the Dual_DDC was implanted in living rats, the DF concentration in blood plasma did not drop significantly in the later periods after implantation relative to that in the early periods before fibrotic tissue formation. When the Dual_DDC was implanted without TR, there was a significant decrease in the blood plasma DF concentration as the time elapsed after implantation. Biopsied tissues around the Dual_DDC exhibited a significant decrease in the fibrotic capsule thickness and collagen density relative to the Dual_DDC without TR, owing to the effect of the local, sustained release of the TR.

## 1. Introduction

For prolonged, controlled drug delivery, implantable devices are often fabricated in the shape of a chip embedded with pairs of a microchannel and drug reservoir serving as a drug diffusion barrier and depot, respectively (Lee et al., 2012; Yang et al., 2018a,b). Thus, after implantation, these devices can deliver drugs in a highly controlled manner for an extended duration. The delivery is mainly modulated by the channel dimensions, and the corresponding regimen is generally considered as advantageous for the delivery of drugs, such as nonsteroidal anti-inflammatory drugs and opioids (Araújo et al., 2009; Martin et al., 2016; Hameed et al., 2020). With the rapid developments in micro- and nano-fabrication technologies (Gardner, 2006; Stevenson et al., 2012), microchannels can be fabricated precisely with high integration, allowing for various drug release profiles in a small-scale implantable device (Hilt & Peppas, 2005; Sutradhar & Sumi, 2016). However, when the devices are implanted in a living body, an inevitable foreign body reaction causes a fibrotic capsule formation around the device, and this additional barrier hampers systemic drug absorption, as reported for many other implantable devices (Bank, 2019; Asrory et al., 2020). This has been considered problematic, as the fibrotic capsule often greatly lowers systemic drug exposure, even for a device with a highly controlled drug release (Ji et al., 2020).

Transforming growth factor-β (TGF-β) is a cytokine secreted during wound healing, and a high expression of TGF-β is closely related to abnormally high fibrosis around an implanted device (Ward, 2008; Meng et al., 2016; Nam et al., 2021). Tranilast (TR) is a drug for inhibiting TGF-β; thus, it has often been prescribed for the treatment of pathological fibrotic diseases in clinical settings (Holmes et al., 2000; Singh et al., 2004). Owing to this effect, the surfaces of medical implants have been coated with TR or a mixture of TR and a polymer for a local, sustained drug release; these combinations have exhibited reduced fibrotic capsules around the implants (Park et al., 2015; Choi et al., 2020). However, the drug or its formulation is often simply dip- or spray-coated. These approaches involve a relatively large initial burst release, leading to inconsistency and a limited duration for the drug release and anti-fibrotic efficacy.

Therefore, for prolonged and systemic drug delivery with minimized effects from fibrotic capsules, we considered an implantable drug-delivery chip (DDC) for the controlled delivery of dual drugs. To test this strategy, we employed TR and diclofenac (DF) for local fibrosis suppression and continuous systemic exposure, respectively. Each drug was loaded in a drug reservoir to be connected to a microchannel for controlled and sustained drug release. However, the TR was exposed in a small dose, so as to be effective locally around the implanted device. DF, as the main drug, was released in a relatively large amount and could be exposed to the blood stream through a reduced fibrotic capsule, thereby prolonging the reproducible and systemic delivery of this anti-inflammatory drug.

The DDC was prepared by assembling two distinct circular chips, each of which was designed to release TR or DF (i.e. TR_DDC and DF_DDC, respectively). The DDC herein was made of a biocompatible polymer, poly(methyl methacrylate) (PMMA), in which a pair of a microchannel and drug reservoir were fabricated by etching with a CO_2_ laser (Lee et al., 2012, 2014). The microchannel was filled with a water-soluble, biocompatible polymer, i.e. polyethylene glycol (PEG), and was connected to a drug reservoir filled with a fine powder of TR or DF. In this way, a bodily fluid, that is, water, could infiltrate via the microchannel to reach a drug reservoir and dissolve the drug. Then, the dissolved drug could diffuse through the same channel for a controlled drug release.

In this study, we attempted to achieve both the rapid onset and sustained release of TR and DF, respectively. For this, we tested the *in vitro* drug release profile with the DDC embedded with a single pair of a drug reservoir and microchannel, with varied lengths of the microchannel for each drug. Based on these results, we selected an appropriate pair of a microchannel and drug reservoir and created a DDC consisting of multiple such pairs to meet the designated doses of TR and DF, respectively. Two distinct DDCs, each for TR or DF delivery, were assembled to prepare a final DDC for dual drug delivery (i.e. the Dual_DDC). To assess the effect of the local fibrosis suppression on the systemic drug exposure, the Dual_DDC was implanted subcutaneously in Sprague-Dawley rats, and the pharmacokinetic profile of the DF was examined and compared with that from the Dual_DDC without TR loading for approximately 30 days. At the end of the experiments, the tissue around the implant was biopsied and analyzed to evaluate the fibrotic capsule formation.

## 2. Materials and methods

### 2.1. Materials

The PMMA plates (thickness = 2 mm) and PEG (average MW = 6 kDa) were purchased from ENGP (Incheon, Republic of Korea) and Acros Organics (Geel, Belgium), respectively. The DF (99.9% purity), TR (98% purity), phosphoric acid, acetonitrile, Tween^®^ 80, ammonium bicarbonate, and formalin (neutral buffered, 10%) were obtained from Sigma (St. Louis, MO). Intravenous catheters and ethylenediaminetetraacetic acid (EDTA) tubes were purchased from BD Biosciences (Franklin Lakes, NJ). Ethanol and ammonium acetate were purchased from Daejung Chemicals (Siheung, Republic of Korea). Betadine was obtained from Green Pharmaceutical (Seoul, Republic of Korea). Phosphate-buffered saline (PBS; pH 7.4) and Ideal 9144 masking tapes were obtained from the Seoul National University Hospital Biomedical Research Institute (Seoul, Republic of Korea) and American Biltrite (Lowell, MA), respectively. Medical epoxy (EPO-TEK^®^ 301-2) was obtained from Epoxy Technology (Billerica, MA).

### 2.2. Drug-delivery chip fabrication

**Scheme 1.**
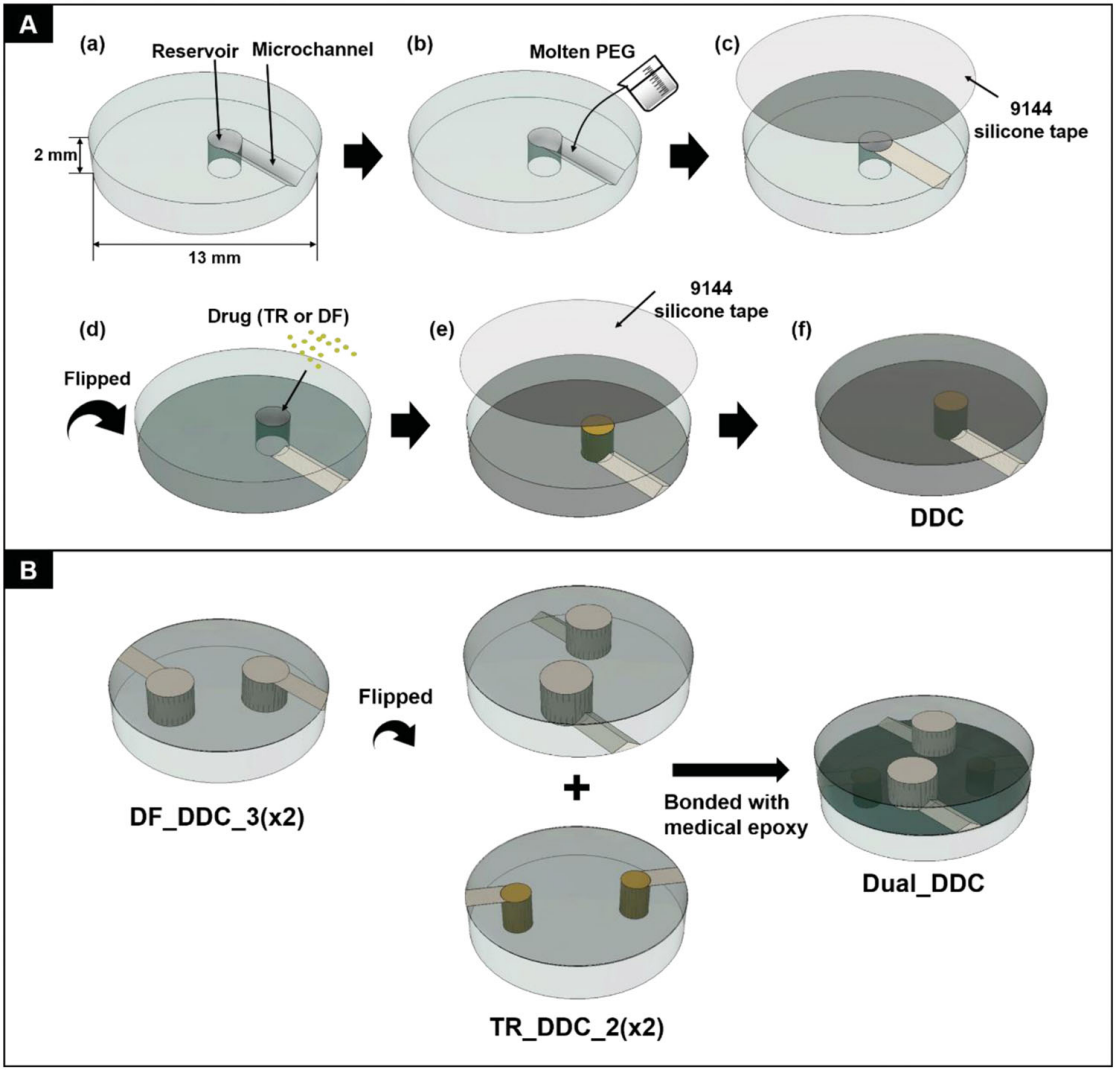
Schematic description of the (A) drug-delivery chip (DDC) and (B) Dual_DDC fabrication.

The DDC was prepared on a PMMA plate (13 mm in diameter and 2 mm in thickness). First, a single microchannel and drug reservoir pair was prepared using a CO_2_ laser (Universal Laser ILS12.150D, Seoul, Republic of Korea) (Scheme 1(A(a))). To prepare the microchannel, a plate was etched at a scanning speed and laser power of 1095 mm/s and 96 W, respectively. We varied the length of the microchannel to determine the designated release profile for TR or DF. Thus, for TR_DDC, the channel length was varied to 2 and 3 mm to produce TR_DDC_2 and TR_DDC_3, respectively. For DF_DDC, the channel length was varied to 2, 3, and 4 mm to produce DF_DDC_2, DF_DDC_3, and DF_DDC_4, respectively. For all microchannels, the cross-sectional area was fixed at 0.37 ± 0.03 mm^2^ and the length was defined as the one measured through the midline in each channel. A drug reservoir was etched at a scanning speed and laser power of 228.6 mm/s and 72 W, respectively, to prepare a cylindrical see-through hole. As a greater amount of DF was needed for systemic exposure, the drug reservoir of the DF_DDC was larger than that of the TR_DDC. Thus, the drug reservoirs were 1.82 and 2.85 mm in diameter for the TR_DDC and DF_DDC, respectively, with the same height of 2 mm. The microchannel was densely filled with molten PEG at 80 °C, which was then solidified at room temperature for 6 h (Scheme 1(b)). Subsequently, the top side of the DDC, where the microchannel was formed, was sealed with a biocompatible Ideal 9144 silicone tape (Scheme 1(A(c))). The DDC was then flipped to expose the bottom side to fill the drug reservoir with a fine power of TR or DF using the doctor blade method (Scheme 1(A(d))), which was then sealed with an Ideal 9144 silicone tape (Scheme 1(A(e))). A DDC with multiple pairs of microchannels and reservoirs was also fabricated using the same process. To produce a DDC for dual drug delivery (i.e. Dual_DDC), two distinct DDCs for TR and DF delivery were assembled together by bonding their bottom sides with medical epoxy (Scheme 1(B)). For comparison, a Dual_DDC without TR loading (that is, a Dual_DDC w/o TR) was also prepared, where the drug reservoir for the TR loading was intentionally left empty.

### 2.3. Characterizations

The morphology of the microchannel filled with PEG was examined using scanning electron microscopy (SEM; JSM-7800 F Prime, Jeol, Akishima, Japan). For this, the microchannels, either empty or filled with PEG, were each placed on a sample mount and sputter-coated with platinum for 60 s (108 auto, Cressington Scientific, Watford, UK). To measure the drug loading amount, the drug-loaded DDC, in which the reservoir was not yet sealed, was fully immersed in the solvent to dissolve the drug. For the TR, the TR_DDC was immersed in 50 ml of PBS (pH 7.4) containing Tween^®^ 80 (1% v/v). For the DF, the DF_DDC was immersed in 20 ml of deionized water. To measure the TR content, the aliquot was assayed using high-performance liquid chromatography–mass spectrometry (HPLC/MS; Agilent 6120 Quadrupole LCMS systems, Agilent Technologies, Santa Clara, CA). The chromatographic separation was performed using a Diamonsil C18 column (4.6 × 150 mm, 5 μm-pore, Dikma, Lake Forest, CA) with a mobile phase pumped at a rate of 0.5 ml/min (Yang et al., 2018a,b). The mobile phase was prepared by mixing acetonitrile and 10 mM of ammonium bicarbonate solution (v/v = 50:50), and the injection volume of the sample was 10 μl. The UV absorbance was measured at 360 nm, and the selective ion monitoring (SIM) ion of the TR was 326 *m/z*. To measure the DF content, the aliquot was assayed spectrophotometrically at 276 nm using a UV–vis spectrophotometer (UV-1800, Shimadzu, Kyoto, Japan) (Lee et al., 2014). All experiments were performed with at least three distinct TR_DDC and DF_DDC samples.

### 2.4. *In vitro* drug release study

The drug-loaded DDCs were each immersed in 50 ml of PBS (pH 7.4) containing Tween^®^ 80 (1% v/v), which was then incubated at 37 °C under 100-rpm agitation in a shaking incubator (SI-600R, Jeio Tech, Seoul, Republic of Korea). At predetermined periods, 25 ml of the release medium was extracted, and an equal volume of fresh medium was added. With this relatively large release medium, we could maintain a good sink condition of TR, which would possess the solubility of about 0.5 mg/ml in PBS (Martin et al., 2012). The obtained media were assessed as described above to measure the concentration of TR or DF.

### 2.5. Cytotoxicity test

To examine the cell compatibility, a DDC without drug loading was utilized to perform an indirect cytotoxicity test (Jeong et al., 2018; Ji et al., 2020). For this, we used L929 mouse fibroblast cells (KLCB, Republic of Korea) grown in an RPMI 1640 medium supplemented with 10% fetal bovine serum, 100 units/ml penicillin, and 100 μg/ml of streptomycin. The DDC was sterilized with ethylene oxide, immersed in 4 ml of the cell culture medium, and incubated at 37 °C in a humidified atmosphere with 5% CO_2_ for 30 days. At scheduled times, the medium was fully collected, and the same volume of fresh medium was added. The collected media were then evaluated using an EZ-Cytox cell viability assay kit (Daeillab Service, Seoul, Republic of Korea). Briefly, 1.0 × 10^5^ cells were seeded in a 96-well plate and incubated for 24 h at 37 °C in a humidified atmosphere with 5% CO_2_. Then, 100 μl of the collected medium was added to each well and incubated for another 24 h. After that, 100 μl of the medium in each well was replaced with an equal volume of fresh medium, and 10 μl of EZ-Cytox reagent was added and incubated for 2 h. The plate was then assessed by measuring the absorbance at wavelengths of 450 nm and 600 nm using a microplate reader (SpectraMax 190 Microplate Reader; Molecular Devices, San Jose, CA). The cell viability was calculated using the following equation: cell viability (%)=(absorbance at 450 nm of the treated well – absorbance at 600 nm of the treated well)/(absorbance at 450 nm of the untreated control well – absorbance at 600 nm of the untreated control well)×100 (Chun et al., 2020).

### 2.6. *In vivo* evaluation

#### 2.6.1. Dual_DDC implantation

For the *in vivo* evaluation, the Dual_DDC was subcutaneously implanted in Sprague-Dawley rats, each aged 8 weeks and weighing 230–280 g. All *in vivo* studies were approved by the Institutional Animal Care and Use Committee (IACUC no. 20-0147-S1A2) at Seoul National University Hospital Biomedical Research Institute. To assess the effects of the TR delivery, the animals were divided into two distinct groups: (1) animals implanted with a Dual_DDC and (2) animals implanted with a Dual_DDC without TR loading (Dual_DDC (w/o TR)). The detailed implantation procedure is shown in Figure S1 in the Supplementary Information.

**Figure 1.**
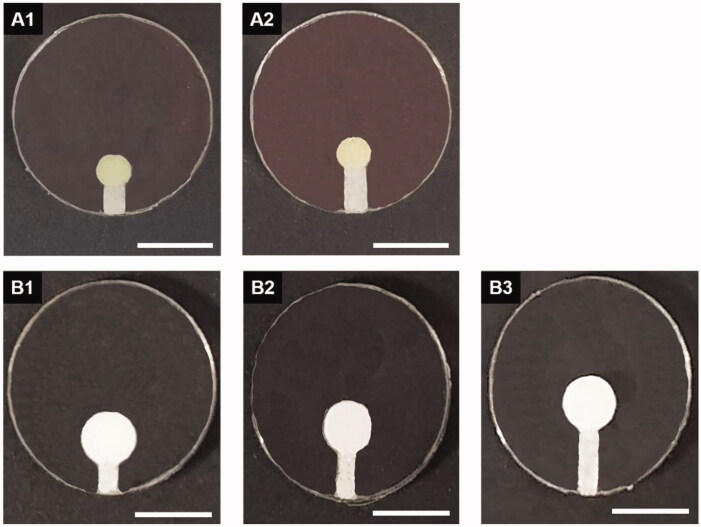
Optical images of the DDC prepared with a single pair of a drug reservoir and microchannel (the latter being of varied lengths). (A1) TR_DDC_2 (2-mm channel), (A2) TR_DDC_3 (3-mm channel), (B1) DF_DDC_ 2 (2-mm channel), (B2) DF_DDC_3 (3-mm channel), and (B3) DF_DDC_4 (4-mm channel). The scale bars are 5 mm.

#### 2.6.2. *In vivo* pharmacokinetic study

We performed a pharmacokinetic analysis for 30 days while the Dual_DDCs with and without TR were implanted. At scheduled times after implantation, 1 ml of blood was collected in an EDTA tube from the lateral tail vein. The collected sample was immediately centrifuged at 12,225×*g* for 10 min to separate the plasma, which was then stored at −20 °C prior to analysis. For measurement of the TR and DF plasma concentrations, 400 μl of methanol was added to 100 μl of thawed plasma to precipitate the proteins. The mixture was vortexed and centrifuged at 12,225×*g* for 10 min. Finally, the supernatant was collected and analyzed using HPLC/MS. For the TR, the measurement conditions were the same as those described above for the *in vitro* drug release study. For the DF analysis, the mobile phase was composed of methanol and 10 mM ammonium acetate (70:30, v/v) (Ji et al., 2020). The flow rate and injection volume were set at 0.5 ml/min and 10 μl, respectively. The UV absorbance was measured at 276 nm, and the SIM ion of the DF was 294 *m/z*.

#### 2.6.3. Histopathology

For the histopathological evaluation, the animals were sacrificed by CO_2_ asphyxiation 30 days after implantation, and the dorsal region tissue around the implanted device was harvested. The biopsied tissue was then fixed in a formalin solution and embedded in paraffin to prepare a block, which was sectioned into 4 μm thick slices. The tissue slices were then mounted on glass slides, which were deparaffinized and rehydrated with xylene and ethanol for staining. To assess the fibrotic capsule thickness and collagen density, hematoxylin and eosin (H&E) and Masson’s trichrome (MT) staining were conducted following a previously reported protocol (Kim et al., 2019). The stained slides were evaluated by a professional pathologist (C. L.) using an optical microscope (ECLIPSE Ts2, Nikon, Minato, Japan). The H&E-stained slide was imaged at ×50 magnification, where the thinnest fibrotic capsule was located and measured. To evaluate the collagen density, the MT-stained tissue in the fibrotic capsule was imaged at a ×200 magnification, and the area of blue-colored collagen was measured as a percentage based on the whole area of the image, using ImageJ (version 1.8.0, National Institutes of Health, Bethesda, MD) (Park et al., 2015). For each group, at least five tissue slide images were randomly selected from each of the five animals; thus, a total of at least 25 images were assessed for statistical analysis on capsule thickness and collagen density.

### 2.7. Statistical analysis

The values of the drug concentration in plasma, capsule thickness, and collagen density were statistically analyzed using the Mann–Whitney *U* test, in which a *p* value of <.05 was considered as statistically significant (SPSS version 26.0, IBM, Armonk, NY).

## 3. Results and discussion

### 3.1. Results

#### 3.1.1. DDC characterizations

In this work, we first prepared the TR_DDC and DF_DDC, each embedded with a single pair of a drug reservoir and microchannel with varied lengths, as shown in Figure 1. According to the SEM images in Figure S2, each microchannel was properly etched with a CO_2_ laser, and could be seamlessly filled with PEG. A reproducible amount of drug was filled in the drug reservoir with the current method, and was measured as 6.63 ± 0.36 and 17.87 ± 0.41 mg per reservoir for the TR_DDC and DF_DDC, respectively.

**Figure 2.**
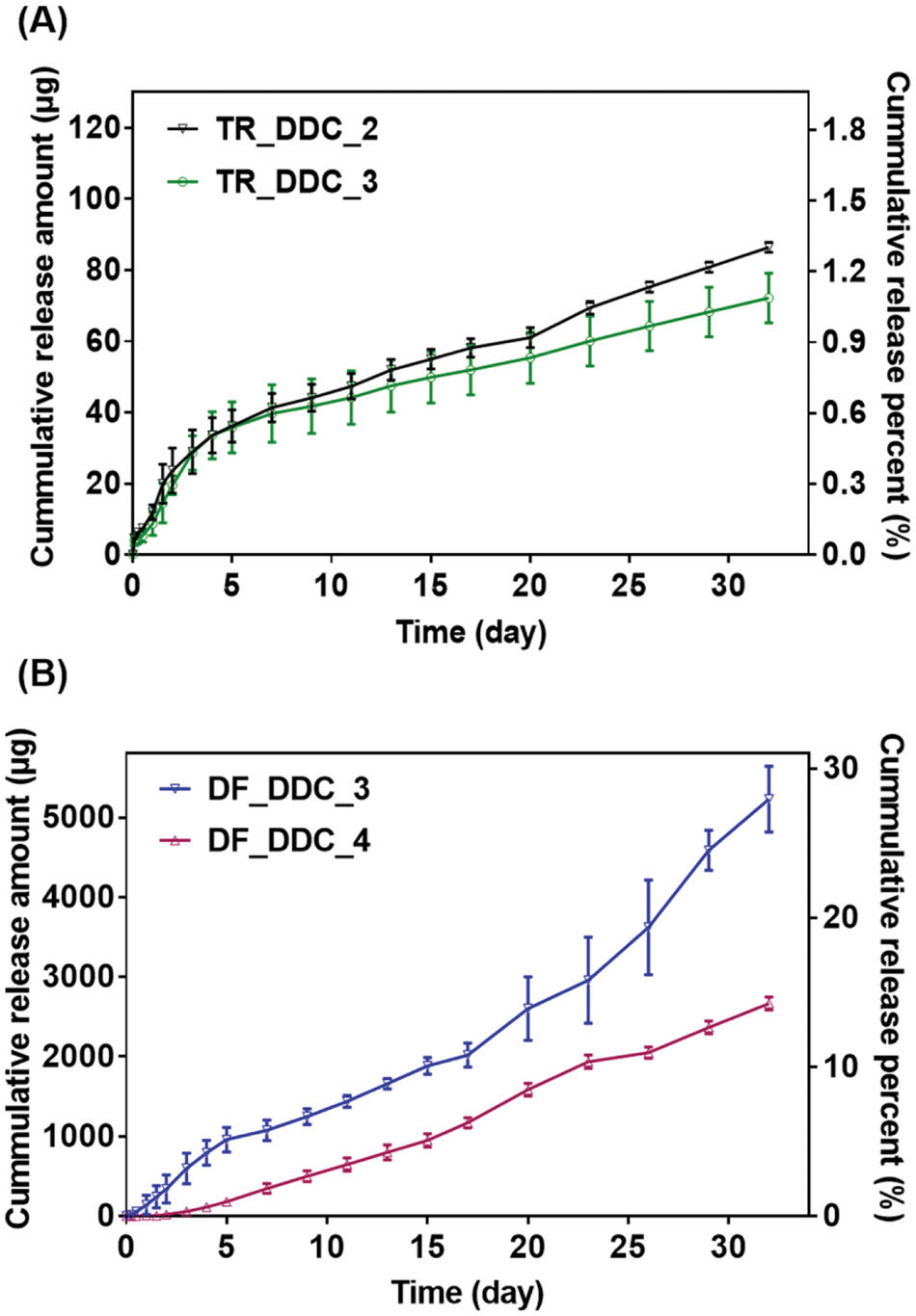
*In vitro* drug release profiles of the (A) TR_DDC and (B) DF_DDC with microchannels of varied lengths. Error bars represent the standard deviation (*n* = 3).

#### 3.1.2. Microchannel selections for dual drug delivery

For the DDC herein, the drug diffusion is modulated by the dimensions of the microchannel, i.e. the drug release is more sustained with a longer channel length (Yang et al., 2013; Marcus et al., 2016). The channel was not left blank but filled with a water-soluble PEG, which would expedite water infiltration by rapid dissolution. However, a long channel delays the onset of the drug release, because to start drug release, a bodily fluid first needs time to infiltrate through the microchannel to reach and dissolve the drug in the reservoir; then, the dissolved drug is diffused out through the same channel. Therefore, in this study, we sought to select a proper microchannel showing both a rapid onset and sustained drug release.

For this, we first performed *in vitro* drug release studies with the TR_DDC and DF_DDC, respectively, each embedded with a single pair of a drug reservoir and microchannel with different lengths. As shown in Figure 2(A), for the TR_DDC, there is a small difference in the TR release pattern between the two different channel lengths of 2 and 3 mm, both of which exhibit a rapid drug-release onset of 2 h. The drug release is faster in the first five days than in the later period; this can be attributed to the effect of the dissolved PEG working as a surfactant to increase the saturation concentration of the poorly water-soluble TR (ca. 0.5 mg/ml) in the reservoir immediately after water infiltration (Heo et al., 2005; Cirri et al., 2007; Martin et al., 2012). After five days, most of the dissolved PEG appears to be washed out from the channel and reservoir, thereby lowering the saturation concentration of the TR, and resulting in slower drug diffusion through the microchannel. Thus, the average release rates of TR for the first five days are 6.92 μg/day (0.10%/day) and 5.90 μg/day (0.09%/day) for the TR_DDC_2 and TR_DDC_3, respectively, and decrease to 1.82 and 1.34 μg/day (0.03 and 0.02%/day) afterwards, respectively.

For the DF_DDC, the effect of channel length on drug release is more prominent, as shown in Figure 2(B). The relatively high water solubility of DF (ca. 5.15 mg/ml) (Kincl et al., 2004) appears to increase the release rate significantly compared with that of TR, even with similar lengths of microchannels. Probably because of this, with a short channel of 2 mm, the drug release is difficult to control, showing an irreproducible profile (Figure S3 in the Supplementary Information). However, the DF is released in a reproducible, sustained manner with the DF_DDC_3 and DF_DDC_4, where the onset time and rate of drug release are more delayed and sustained, respectively, as the channel length increases. Thus, the onset times of the drug release are 12 h and two days for the DF_DDC_3 and DF_DDC_4, respectively, after which the average release rates are 145.64 and 90.59 μg/day (0.81 and 0.51%/day), respectively.

**Figure 3.**
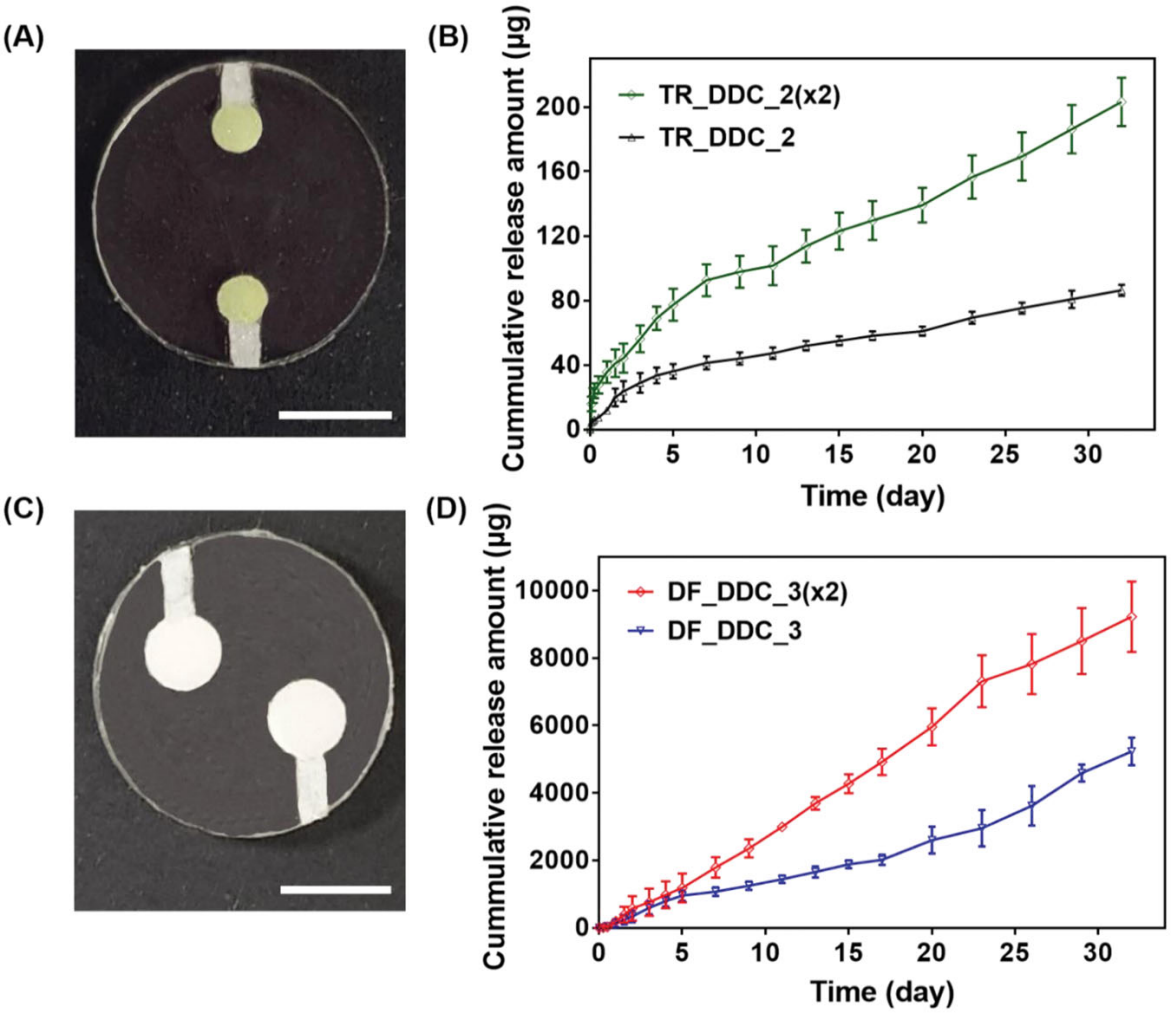
(A) Optical image of TR_DDC_2(x2). (B) *In vitro* TR release profiles of TR_DDC_2(x2) and TR_DDC_2. (C) Optical image of DF_DDC_3(x2). (D) *In vitro* DF release profiles of DF_DDC_3(×2) and DF_DDC_3. (A,C) The scale bars are 5 mm. (B,D) Error bars represent the standard deviation (*n* = 3).

Among the microchannels showing a rapid onset of drug release in hours, as well as a reproducible, sustained drug release, we sought to find the one meeting the designated doses for each of the drugs. For the TR, we set the needed daily dose to be close to 1.54 μg/cm^2^/day, as this dose per unit surface area of the implant appeared to suppress fibrosis in our previous study (Park et al., 2015). In this work, we assembled two DDCs together for dual drug delivery (i.e. the Dual_DDC); this provided a total surface area of 4.28 cm^2^, and thus, our Dual_DDC would need the TR dose to be close to 7 μg/day. According to the results in Figure 2(A), two pairs of a 2-mm microchannel and drug reservoir could meet this dose; thus, we prepared these two pairs in a single DDC to produce a TR_DDC_2(x2) (Figure 3(A)). As shown in Figure 3(B), the average rate of drug release doubles to 6.9 μg/day, i.e. close to our designated dose of TR. The onset of drug release is still at 2 h, as observed with the TR_DDC_2 with a single pair of a microchannel and drug reservoir.

The DF herein was proposed to be systemically exposed, and thus, we pursued the dose to be sufficiently high so that the *in vivo* concentration of the DF in the blood plasma could be measurable via HPLC/MS, as described above. For subcutaneously implanted devices in a previous study (Ji et al., 2020), a daily DF dose of over 180 μg/day was seen to be detectable using the current animal model and assay method. According to the results in Figure 2(B), two pairs of a 3-mm microchannel and reservoir could meet this dose; thus, we again prepared these two pairs in a single DDC to produce a DF_DDC_3(x2) (Figure 3(C)). Figure 3(D) shows the *in vitro* drug release profile with the DF_DDC_3(2x), which exhibits a doubled average rate of the DF release amount (302.17 μg/day), with the same onset time as that observed with the DF_DDC_3 possessing a single pair of the microchannel and reservoir.

**Figure 4.**
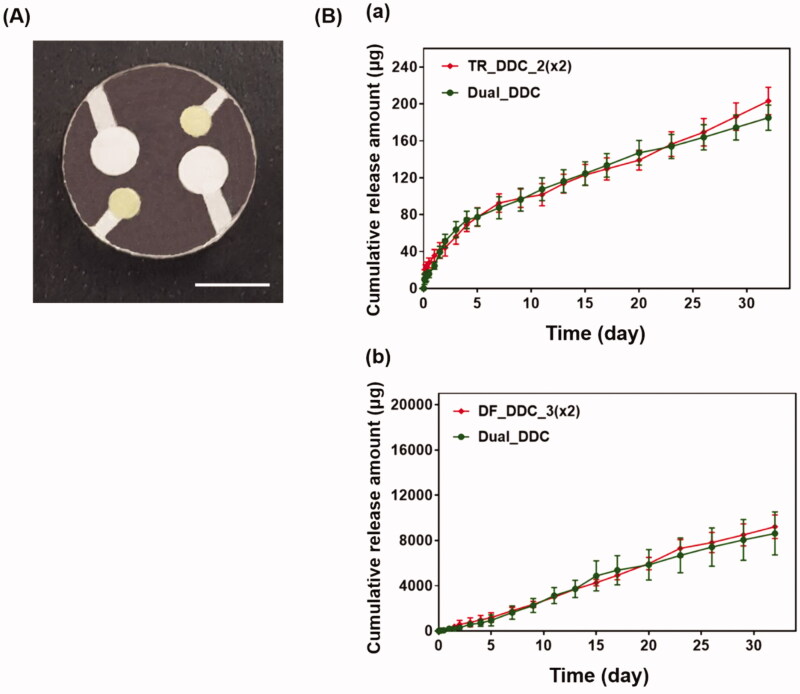
(A) Optical image of the Dual_DDC. The scale bar is 5 mm. (B) *In vitro* release profiles of (a) TR and (b) DF with the Dual_DDC, which were compared with that of TR_DDC_2(×2) and DF_DDC_3(x2), respectively. Error bars represent the standard deviation (*n* = 3).

**Figure 5.**
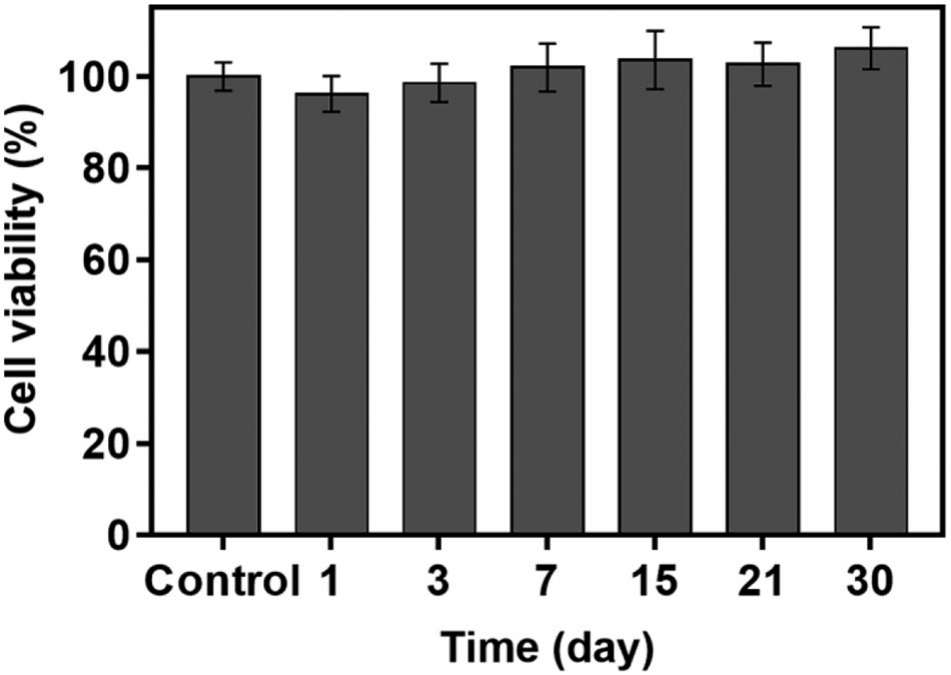
Cytotoxicity evaluation of the blank Dual_DDC without drugs using L929 fibroblasts. Error bars represent the standard deviation (*n* = 3).

Finally, we bonded the TR_DDC_2(x2) and DF_DDC_3(x2) together to produce a single integrated DDC, that is, the Dual_DDC (Figure 4(A)), as depicted in Scheme 1(B). Aiming to not affect the release of each drug, the microchannels and reservoirs were arranged so as to not overlap each other, and so that each opening for drug release could be placed apart. Figure 4(B) shows the *in vitro* release profiles of TR and DF with the Dual_DDC, which are almost identical to those observed for the TR_DDC_2(x2) and DF_DDC_3(x2), respectively. This result also suggested that two different drugs in the same release medium did not interact each other when measured with the methods employed in this work. When the blank Dual_DDC (i.e. the dual_DDC without drug loading) was tested with the L929 fibroblasts, there was no cytotoxicity, indicating that almost no toxic compounds leaked from the body of the Dual_DDC (Figure 5). This result was expected, as in this work, the DDC comprised only biocompatible materials (Gautam et al., 2012; Paz et al., 2021).

#### 3.1.3. *In vivo* evaluation

**Figure 6.**
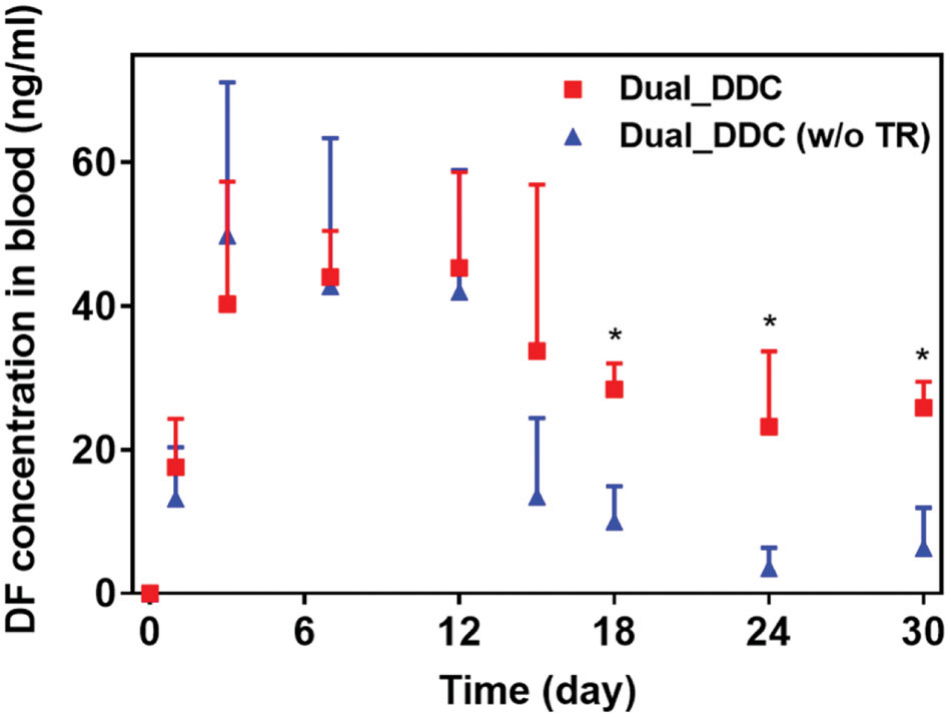
*In vivo* pharmacokinetic profiles of DF with the Dual_DDC (■), which was compared with that of the Dual_DDC without TR (i.e. the Dual_DDC (w/o TR) (▲)). Error bars represent the standard deviation (*n* = 5). *Statistically significantly different between two groups (*p*<.05).

To assess the *in vivo* effects of the dual delivery of TR and DF, we subcutaneously implanted the Dual_DDC in living rats, and the pharmacokinetic profile of the DF was examined for 30 days after implantation. For comparison, animals in a control group were implanted with a Dual_DDC (w/o TR), that is, a Dual_DDC containing DF only without TR. As shown in Figure 6, for both animal groups, DF is continuously detected for all testing periods, indicating a sustained release of DF sufficient for exposure to the blood stream. The plasma concentration of DF does not differ between the Dual_DDC and Dual_DDC (w/o TR) in the early periods after implantation, until 12 days.

However, the difference in the blood plasma drug concentration becomes apparent from day 15, where a substantially low amount of DF is detected with the Dual_DDC (w/o TR). The range of average plasma drug concentration drops to 3.5–13.5 ng/ml, i.e. less than a quarter of the concentration observed during the early periods after implantation. In contrast, for the Dual_DDC loaded with TR, the decrease in the plasma drug concentration is not significant. The drug is detected at concentrations ranging from 23.2 to 33.8 ng/ml, i.e. still comparable to those obtained in the early periods. Notably, this blood plasma drug concentration is more than 2.8 times higher than that of the Dual_DDC (w/o TR). The blood concentration of TR with the Dual_DDC is very low (<9 ng/ml) for all testing periods, as we intentionally set the TR release at a low dose to be effective locally around the implant (Figure S4 in the Supplementary Information). As expected, no TR is detected with the Dual_DDC (w/o TR).

**Figure 7.**
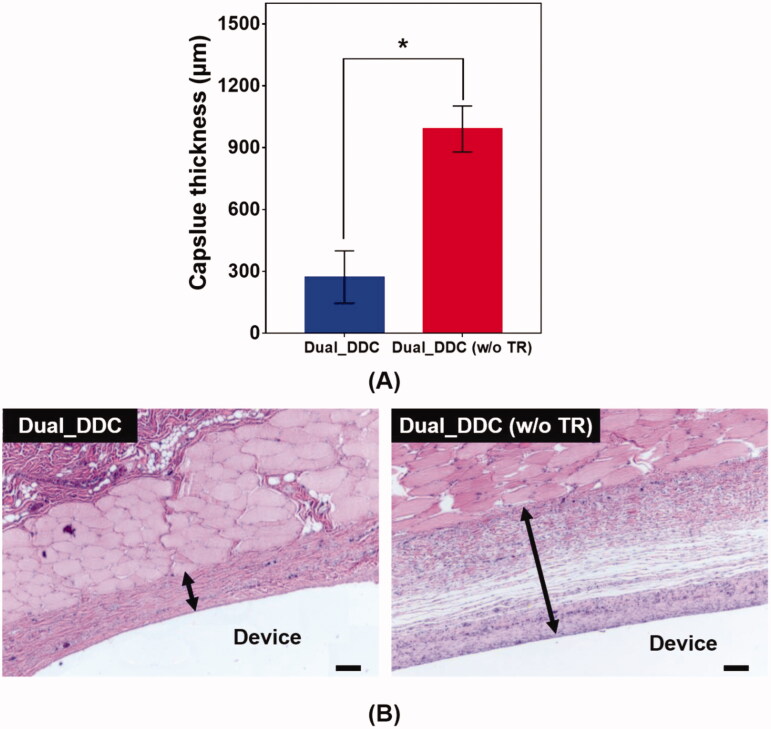
Evaluation of (A) capsule thickness and (B) representative images of the hematoxylin and eosin (H&E)-stained tissues biopsied 30 days after DDC implantation. Error bars represent the standard deviation (*n* = 25). *Statistically significantly different between Dual_DDC and Dual_DDC (w/o TR) (*p*<.05). The double-ended arrow indicates the capsule thickness. The scale bars are 200 μm.

**Figure 8.**
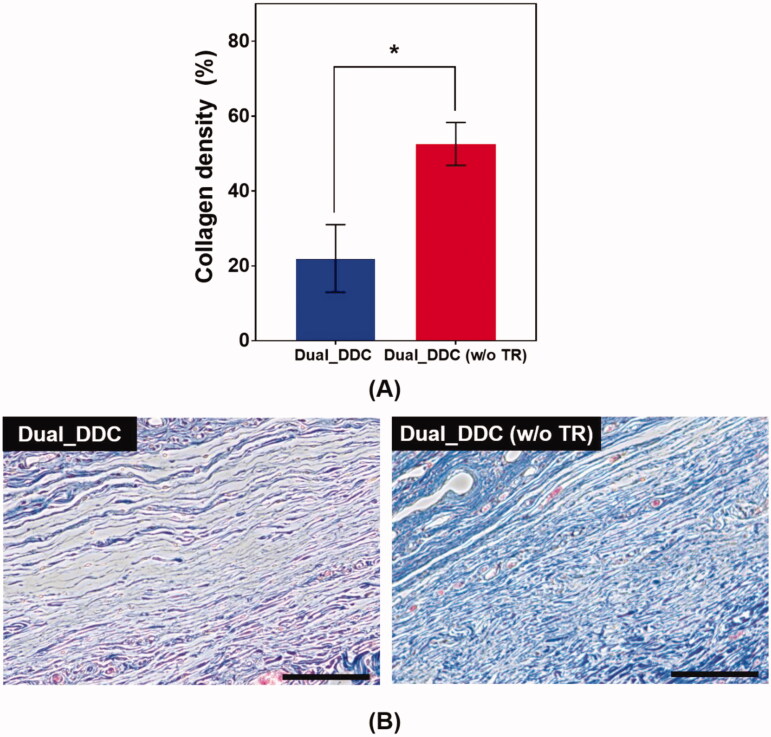
Evaluation of (A) collagen density and (B) representative images of Masson’s trichrome (MT)-stained tissues biopsied 30 days after DDC implantation. Error bars represent the standard deviation (*n* = 25). *Statistically significantly different between Dual_DDC and Dual_DDC (w/o TR) (*p*<.05). The scale bars are 200 μm.

To further confirm the antifibrotic effect of the TR, we performed a histopathological analysis of the tissues around the Dual_DDC and Dual_DDC (w/o TR), as biopsied at the end of the experiments. As shown in Figure 7, the capsule thickness around the Dual_DDC is significantly thinner than that of Dual_DDC (w/o TR), with values of 273.0 ± 127.2 μm and 990.9 ± 111.5 μm, respectively. Moreover, in these thin capsules, the density of the collagen also decreases more significantly with the Dual_DDC than with that of the Dual_DDC (w/o TR) (Figure 8). Both results indicate that a sustained release of TR indeed inhibits the fibrotic capsule formation around the Dual_DDC, leading to a weaker barrier against the DF exposure to the blood stream, and thus, a higher DF concentration in the blood than that of the Dual_DDC (w/o TR) (Figure 6).

### 3.2. Discussion

To enable long-term drug delivery with high precision, many implantable devices have been developed with sophisticated designs (Elman et al., 2009; Meng & Hoang, 2012), and some of them have already been approved for clinical use (Pons-Faudoa et al., 2019). Unlike biomaterial-based formulations, these devices deliver drugs in a highly controlled and even timed manner; the delivery is mediated by accurate geometric factors, as well as by mechanical modalities (Sanjay et al., 2018). However, to possess these advantageous features along with the complex structures, the devices must inevitably become slightly bulky and nondegradable, often leading to a drug diffusion barrier of fibrotic tissues surrounding the implant (Carnicer-Lombarte et al., 2012; Robotti et al., 2020). Therefore, although highly controlled in *in vitro* environments, the drug release profile *in vivo* is not reproducible, and often shows much lower systemic drug exposure in later periods after implantation (Lee et al., 2018).

To overcome this limitation, we proposed a strategy of co-delivery of dual drugs: one was a main-purpose drug (DF) to be exposed systemically, and the other was an anti-fibrotic drug (TR) for local delivery around the implant. In this work, we assessed this strategy using a DDC embedded with microchannels as an example of a nondegradable, implantable device for precise drug delivery. We utilized the channels in millimeter length to achieve both relatively rapid onset and sustained drug release according to our previous reports (Lee et al., 2014; Ji et al., 2020). Our findings revealed that the microchannels could modulate the release pattern of each drug in a controlled manner, depending on their dimensions (Figure 2). Therefore, the controlled delivery of dual drugs, each with a designated dose, could be achieved by a simple integration of multiple pairs of channels and drug reservoirs in a single Dual_DDC (Figures 3 and 4). When tested *in vivo*, the effect of the TR release was evident, showing thinner and weaker fibrotic capsules (Figures 7 and 8) and hence, a higher systemic exposure of DF compared with that of the Dual_DDC (w/o TR) (Figure 6). As we pursued the TR to be effective mainly at the local tissues around the implant, the dose was modulated to be comparably low; thus, the systemic exposure of TR was observed as minimal (Figure S4).

Our findings also revealed that a fibrotic capsule, albeit quite diminished, was formed around the nondegradable Dual_DDC, which might not be fully prevented owing to an inflammatory response essential for wound healing (Diegelmann & Evans, 2004). Due to this, the DF concentration in the blood from 15 days after implantation was still lower than that in the early periods when the capsule was not yet formed. However, the decrease in drug plasma concentration was only approximately a quarter (Figure 6). With this small difference, the current Dual_DDC would be able to establish an appropriate release rate to maintain the plasma drug concentration within the required therapeutic window, both in the early and later periods after implantation, as the maximum allowable dose of DF is three times higher than the minimum effective dose in clinical settings (Odom et al., 2014). As the drug release rate depended mainly on the dimensions of the microchannel in the Dual_DDC, the period until complete drug release could be modulated simply by varying the size of the drug reservoir, that is, the initial drug loading amount. The prototype of the Dual_DDC would release DF for up to four months (0.84%/day) (Figure 4). During this period, the fibrotic capsule (Figures 7 and 8) would be stabilized without significant growth (Farra et al., 2012; Park et al., 2015) and thus, the level of DF systemic exposure in our current results was expected to be maintained. Moreover, as the TR would be released continuously together (0.05%/day) (Figure 4), it was expected to inhibit any further deposition of fibrotic tissues.

## 4. Conclusions

We suggest an implantable DDC embedded with pairs of a microchannel and drug reservoir for the controlled delivery of dual drugs, i.e. a main-purpose drug for systemic exposure, together with an antifibrotic drug for suppression of local fibrotic capsule formation. For each drug, a proper selection of the microchannel length allows the DDC to start the drug release within hours, and the drug can be released in a sustained manner for prolonged periods. The required dose of the drug release rate can also be obtained by integrating multiple pairs of a microchannel and drug reservoir in a single DDC. Therefore, a DDC for dual drug delivery can be prepared by assembling two separate DDCs for the delivery of each drug. Our *in vivo* experimental results reveal that a Dual_DDC loaded with both DF and TR indeed suppresses local fibrosis; thus, there is no prominent difference between the DF concentrations in the blood plasma at the early and later periods after implantation. Therefore, we conclude that the co-delivery of an anti-fibrotic drug such as TR using a microchannel-based implantable DDC can be a useful strategy for more prolonged and reproducible systemic delivery for drugs of interest.

## Supplementary Material

Supplemental Material
