# Supplementary material for: Microchannel-embedded implantable device with fibrosis suppression for prolonged controlled drug delivery
Source: Drug Deliv. 2022 Feb 11;29(1):489–98. doi: 10.1080/10717544.2022.2032873 (PMC8843219; doi:10.1080/10717544.2022.2032873)
Supplement: Supplemental Material [file IDRD_A_2032873_SM5823.docx]

**SUPPLEMENTARY INFORMATION**

**Microchannel-Embedded Implantable Device with Fibrosis Suppression for Prolonged Controlled Drug Delivery**

Han Bi Ji, Jae Young Hong, Cho Rim Kim, Chang Hee Min, Jae Hoon Han, Min Ji Kim,

Se-Na Kim, Cheol Lee and Young Bin Choy

**
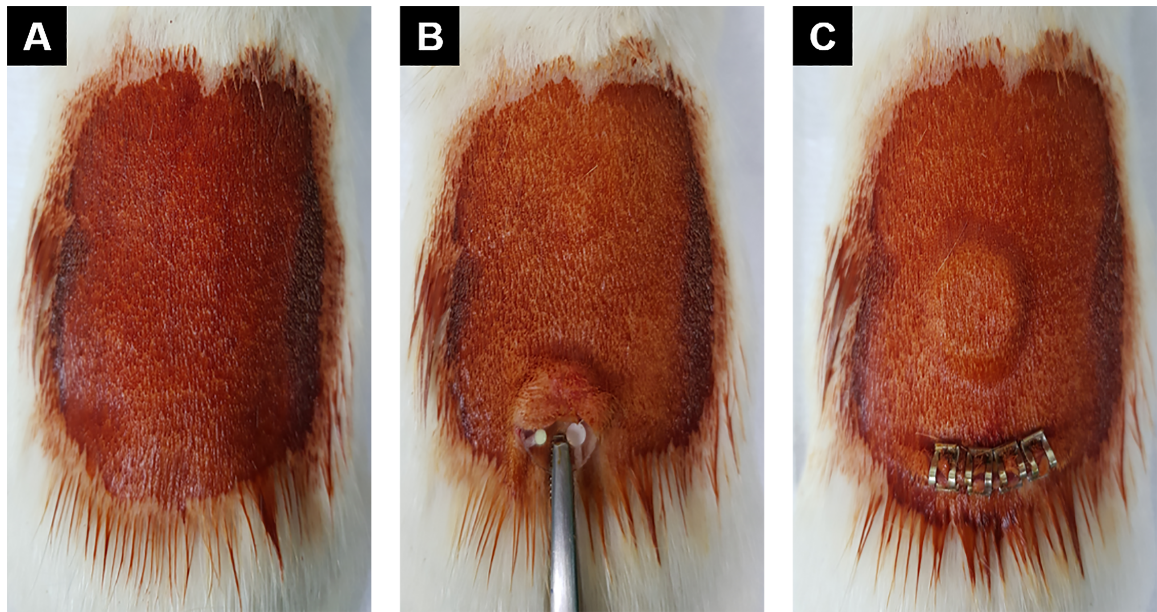
**

**Figure S1.** Surgical procedure for the implantation of the dual drug-delivery chip (Dual_DDC). For this procedure, rats were anesthetized by isoflurane inhalation. (A) The dorsal area was shaved and sterilized with betadine. (B) The Dual_DDC was implanted into the subcutaneous pocket. (C) The wound was closed using surgical clips (9 mm Autoclips, MikRon Precision, CA, USA) and disinfected with betadine.

**
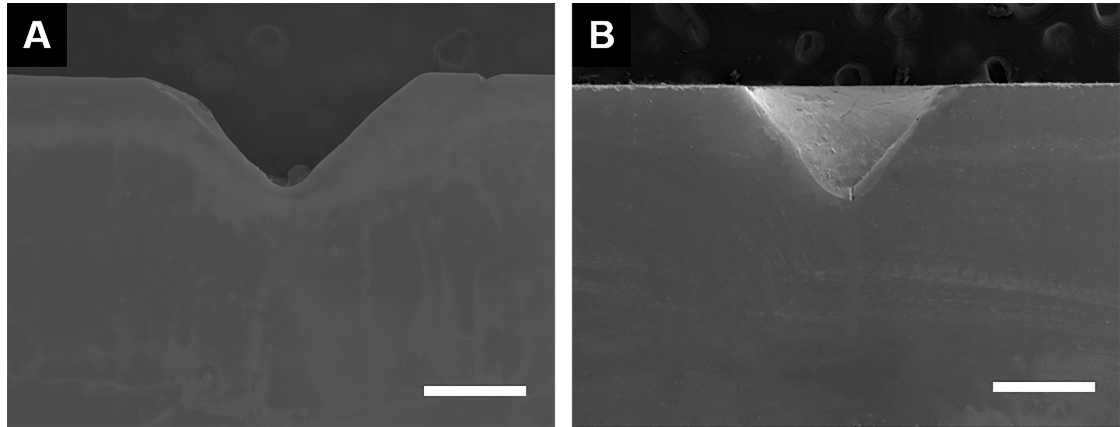
**

**Figure S2.** Scanning electron microscopy (SEM) images of the cross-section of a micro-channel (A) before and (B) after filling with polyethylene glycol (PEG). The scale bars are 500 μm.


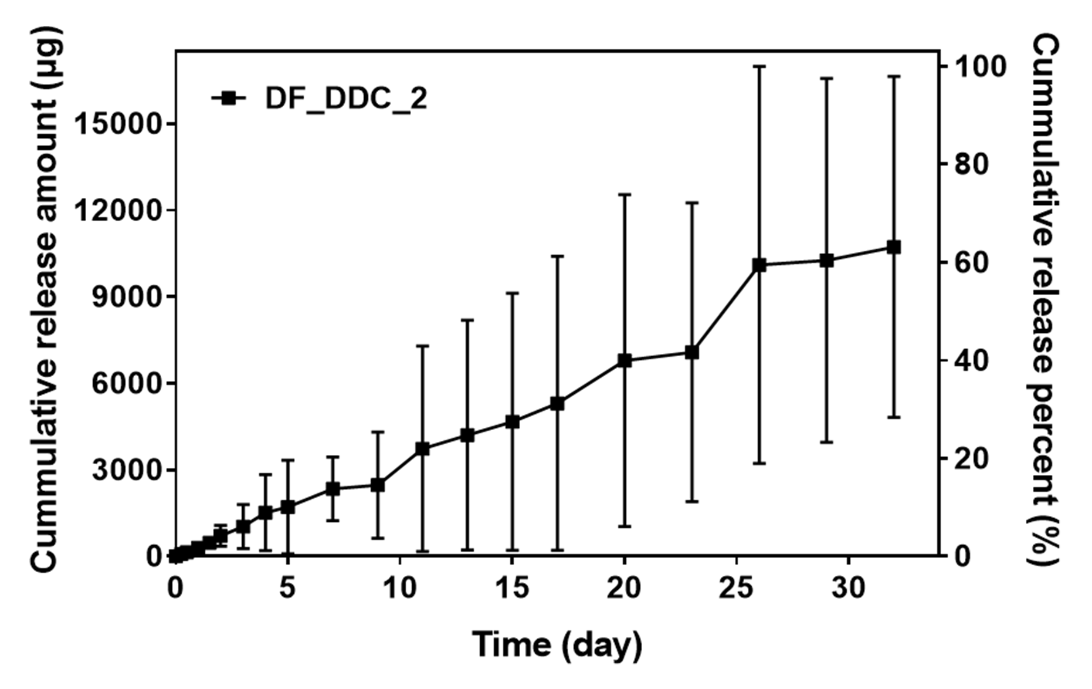


**Figure S3.** *In vitro* release profile of diclofenac (DF) with the DF_DDC_2. Error bars represent standard deviation (n =3); these are very large, indicating very low controllability of the drug release with this short channel.


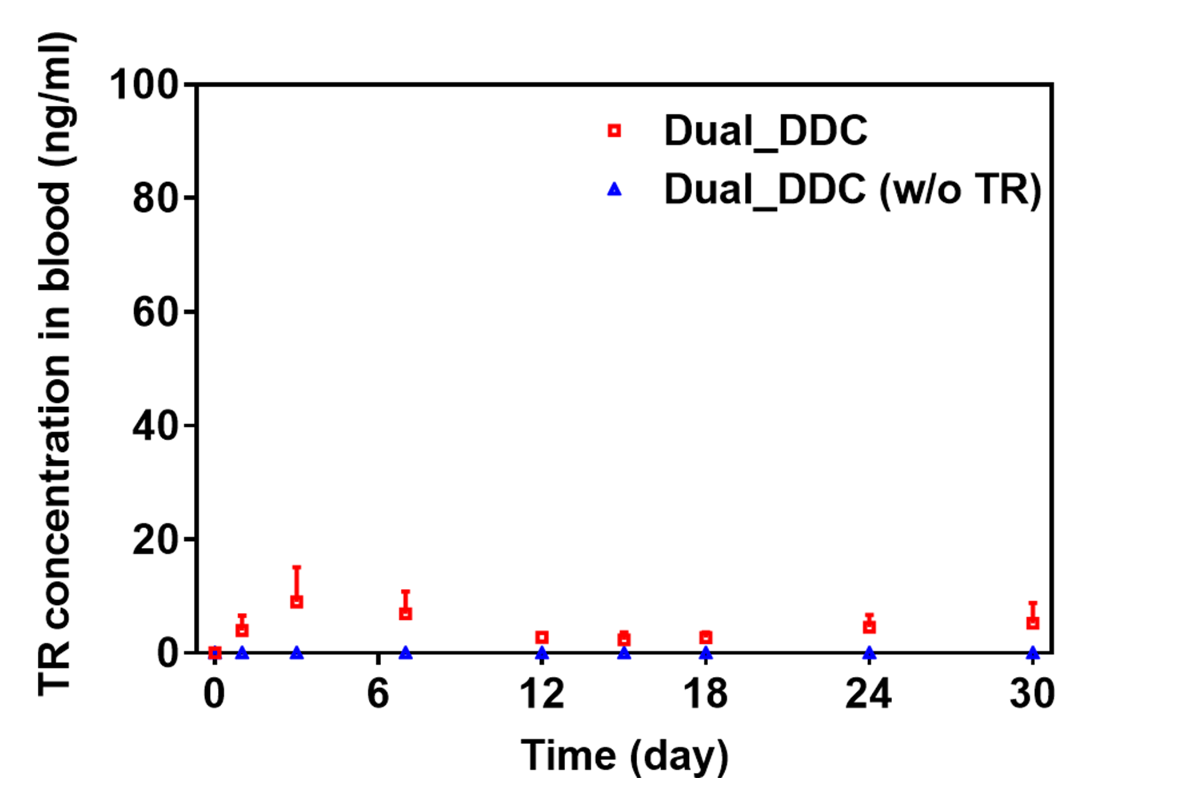
**Figure S4.** Blood plasma concentrations of tranilast (TR) with the Dual_DDC and Dual_DDC (w/o TR). Error bars represent the standard deviation (n = 5).
